# Optimal planting pattern of cotton is regulated by irrigation amount under mulch drip irrigation

**DOI:** 10.3389/fpls.2023.1158329

**Published:** 2023-05-30

**Authors:** Wenqing Zuo, Baojian Wu, Yuxuan Wang, Shouzhen Xu, Jingshan Tian, Xingli Jiu, Hengyi Dong, Wangfeng Zhang

**Affiliations:** ^1^Key Laboratory of Oasis Eco–Agriculture, Xinjiang Production and Construction Corps, College of Agronomy, Shihezi University, Shihezi, Xinjiang, China; ^2^Regimental Farm 149, Division Eight, Xinjiang Production and Construction Corps, Shihezi, China

**Keywords:** mulch drip irrigation, row spacing configuration, cotton, leaf area index, soil water consumption

## Abstract

**Objective:**

It is of great importance to explore agronomic management measures for water conservation and cotton yield in arid areas.

**Methods:**

A four–year field experiment was conducted to evaluate cotton yield and soil water consumption under four row spacing configurations (high/low density with 66+10 cm wide, narrow row spacing, RS_66+10H_ and RS_66+10L_; high/low density with 76 cm equal row spacing, RS_76_H and RS_76_L) and two irrigation amounts (CI:conventional drip irrigation; LI:limited drip irrigation) during the growing seasons in Shihezi, Xinjiang.

**Results:**

A quadratic relationship was observed between the maximum LAI (LAI_max_) and seed yield. Canopy apparent transpiration rate(CAT), daily water consumption intensity (DWCI) and crop evapotranspiration (ET_C_) were positively and linearly correlated with LAI. The seed yields, lint yields, and ET_C_ under CI were 6.6–18.3%,7.1–20.8% and 22.9–32.6%higher than those observed under LI, respectively. The RS_66+10H_ under CI had the highest seed and lint yields. RS_76_L had an optimum LAI_max_ range, which ensured a higher canopy apparent photosynthesis and daily dry matter accumulation and reached the same yield level as RS_66+10H_; however, soil water consumption in RS_76_L was reduced ET_C_ by 51–60 mm at a depth of 20–60 cm at a radius of 19–38 cm from the cotton row,and water use efficiency increased by 5.6–8.3%compared to RS_66+10H_ under CI.

**Conclusion:**

A 5.0<LAI_max_<5.5 is optimum for cotton production in northern Xinjiang, and RS_76_L under CI is recommended for high yield and can further reduce water consumption. Under LI, the seed and lint yield of RS_66+10H_ were 3.7–6.0% and 4.6–6.9% higher than those of RS_76_L, respectively. In addition, high-density planting can exploit the potential of soil water to increase cotton yields under water shortage conditions.

## 1 Introduction

Cotton (*Gossypium hirsutum* L.) is the most widely cultivated and vital fiber crop worldwide (Dai and Dong, 2014). It is mainly planted in the arid areas of China, the United States, Australia, Pakistan, and India (Tian et al., 2017; Tabashnik and Carrière, 2019; Anwar et al., 2020). Insufficiency or deficit irrigation is the main obstacle to the sustainable development of cotton in arid regions (Forouzani and Karami, 2011; Wei et al., 2022). However, agricultural irrigation water accounts for more than 60% of total land water consumption (Qin et al., 2016; Rafiee and Kalhor, 2016). The increasing demand for crop yield resulting from population growth further exacerbates water shortages in arid areas (Neumann et al., 2011). Therefore, it is crucial to ensure that agricultural development measures in arid areas consider the regulation of field management, appropriate irrigation methods to improve the rational use of water resources, and high crop yield.

Augmenting planting density is an important cultivation practice for increasing crop yield (Zhang et al., 2004; Feng et al., 2017), because it can increase leaf area index (LAI) and the interception of light energy, resulting in higher canopy photosynthetic capacity (Zhang et al., 2004; Liao et al., 2022). As the largest cotton-producing region in China, Xinjiang has a favorable ecological environment for producing high quality and yield cotton, due to abundant sunshine, a dry climate, and large diurnal temperature differences (Li et al., 2017). The widespread application of mulch drip irrigation technology since the late 1990s has effectively improved the water resource efficiency of crop production in arid and semi–arid regions in northern China (Dai and Dong, 2014; Guo et al., 2021). In recent years, cotton planting density in Xinjiang has been stable at around 22.5×10^4^ plant hm^–2^ (Hu et al., 2021) due to the breeding of new varieties (Wang et al., 2021a) and the rational use of growth regulators (Shi et al., 2022). A higher LAI combined with sufficient light in Xinjiang improved the effective interception of photosynthetic radiation (Feng et al., 2017; Wu et al., 2017), canopy photosynthesis, and biomass accumulation, reduces the number of bolls per plant, increases the number of bolls per population (Zhang et al., 2004), thus increasing cotton yield (Dong et al., 2006; Araus et al., 2021). Therefore, increasing planting density to improve above–ground LAI is an important measure to obtain high crop yield. However, a higher LAI may lead to mutual shading within the cotton canopy, thus affecting population photosynthetic productivity (Hu et al., 2021; Paul et al., 2021). Appropriate planting density can also improve cotton yield by improving dry matter accumulation and potassium fertilizer absorption (Khan et al., 2017a; Khan et al., 2017b). Studies on cotton LAI in the Americas and other major cotton–growing countries in Asia have shown that the optimum LAI for a higher cotton yield is between 4.0 and 5.0 (Kerby et al., 1990; Heitholt, 1994; Bilal et al., 2019). However, there is still no definite conclusion regarding the optimum cotton LAI range after machine–harvested planting was implemented in Xinjiang.

A high crop yield in arid areas should be accompanied by efficient utilization of water resources. Crop evapotranspiration (ET_C_), which includes soil evaporation and crop transpiration, is a key component of water consumption in agricultural fields, accounting for more than 90% of agricultural water use (Hou et al., 2022). Moreover, a high planting density significantly increases the ET_C_ (Cui et al., 2018; Wang et al., 2021b). Therefore, higher irrigation volumes are required to meet the demand for higher yields under high-density planting (Kodur, 2017; Wu et al., 2017; Hernandez et al., 2021). Some studies have also shown that ET_C_ is related to leaf area, but a larger LAI did not lead to higher soil water consumption because of shading between leaves, although more bare land areas were covered (Rahman et al., 2018; Di et al., 2019). Therefore, it is necessary to adjust the planting density and irrigation amount to regulate the aboveground LAI in a suitable range to improve the photosynthetic rate and increase the dry population accumulation (Yao et al., 2017; Chen et al., 2019). The adoption of new irrigation practices, such as sub–membrane drip irrigation (Zou et al., 2020), deficit irrigation (Paul et al., 2021) and limited irrigation (Chen et al., 2019) are common irrigation practices for improving water use efficiency (WUE) in arid areas. Many scholars have shown that, proper irrigation kept the crop root system in the irrigated wet zone (Chen et al., 2018), improves root morphology and physiological activity (Luo et al., 2014), and facilitates rapid water uptake by the root system for upward transport through the main stem to supply upper ground growth (Chen et al., 2019).

The combination of mulch drip irrigation and high-density planting is an important technical measure for high cotton yields in Xinjiang (Sui et al., 2018; Guo et al., 2021). However, the increasing shortage and unbalanced distribution of water have severely restricted cotton production in this area (Li et al., 2021). Therefore, it is important to explore water saving strategies and high cotton yields by conducting research on agronomic technical measures based on drip irrigation projects. We hypothesize that under water deficit conditions, increasing planting density could maintain the photosynthetic productivity of cotton populations by maximizing the use of soil water and increasing the population LAI to ensure high cotton yields. While under water-sufficient conditions, low-density planting has the potential to optimize canopy LAI to achieve high photosynthetic productivity while reducing soil water consumption. We hypothesized that there would be an optimal planting pattern under different water supply conditions to achieve a combination of water savings and cotton yield. The objectives of this study were (a) to determine the effects of irrigation amount and row spacing configuration on LAI dynamics and population photosynthesis capacity during the cotton reproductive period, and (b) to clarify the population transpiration water consumption and soil water consumption and provide suitable field management measures for high yields and water conservation in arid areas.

## 2 Materials and methods

### 2.1 Study site and experimental design

**Figure 1:**
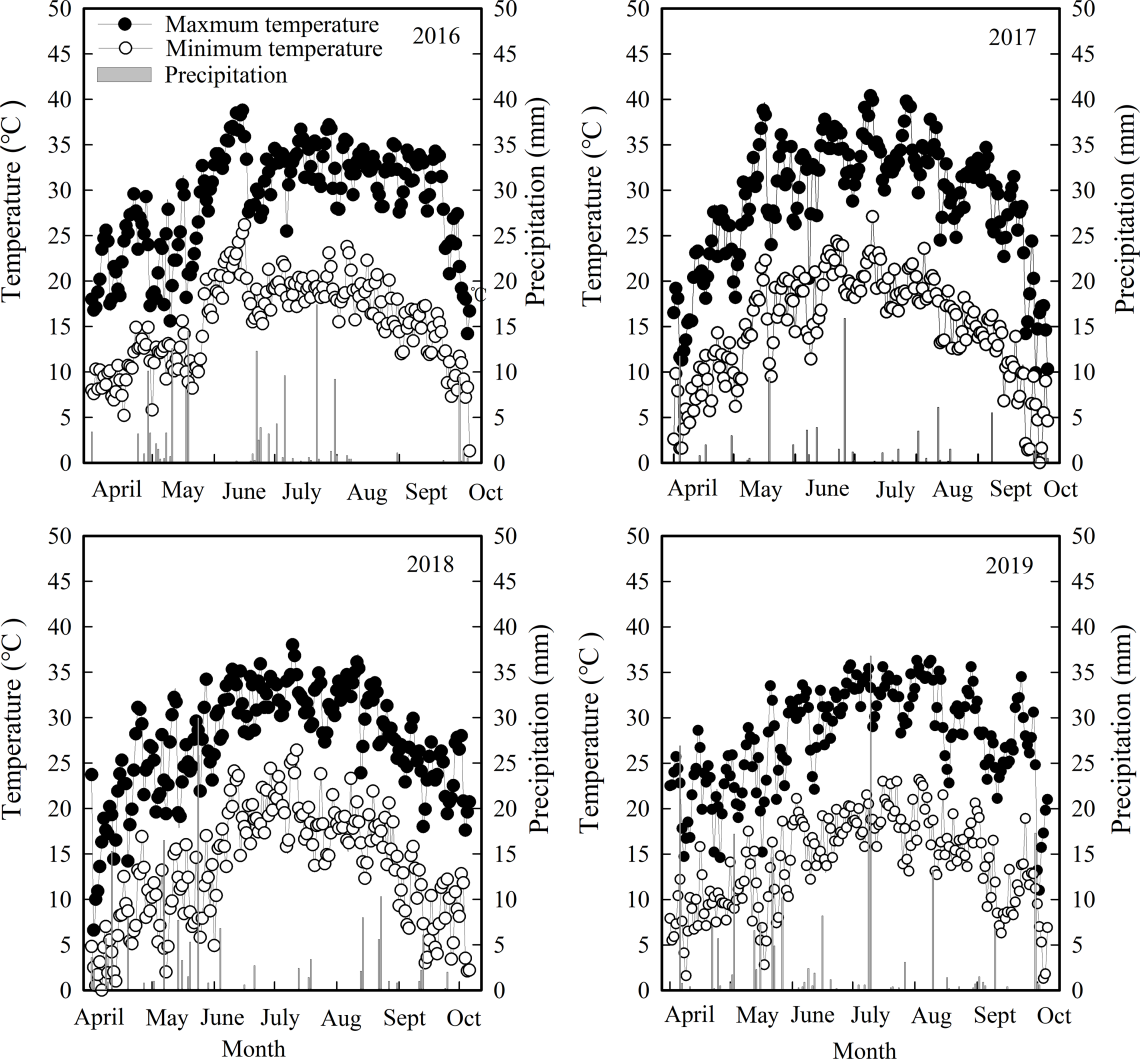
Daily maximum temperature, minimum temperature, and rainfall from planting until harvest (April to October) in 2016, 2017, 2018, and 2019.

A four–year field experiment was conducted at the 13^th^ Company (45°12′N, 86°05′E, 380m a. s. l.) and 11^th^ Company of the 149th Regimental Farm of Sihezi (45°12′N, 86°06′E, 380m, a. s. l.) and the experimental sites of Shihezi University (45°19′N, 86°03′E, 482m a. s. l.) and Wulanwusu Agrometeorological Experiment Station of Shihezi (44°17′N, 85°49′E, 520m a. s. l.) during the 2016–2019 growing seasons, respectively. The four test sites are typical of temperate continental climates. The locations in which this trial was conducted were in accordance with conventional tillage. Weather data for the sites were obtained from the nearest meteorological station. Daily maximum temperature, minimum temperature, and rainfall from planting until harvest (April to October) for 4 years are shown in **Figure 1**. The soil texture, soil moisture content, soil bulk density, and soil nutrient content of the test area before sowing are shown in **Table 1**.

**Table 1:** Soil texture and soil water content before sowing at different test sites used for evaluating the optimal planting pattern of cotton (*Gossypium hirsutum)* regulated by water amount under mulch drip irrigation in Xinjiang, China.

Station	Soil depth (cm)	Soil water content (%)	Bulk density (g· cm^− 3^)	Texture	Alkali-hydro nitrogen (mg·kg^−1^)	Available phosphorus (mg·kg^−1^)	Available potassium (mg·kg^−1^)	Organic matter (g·kg^−1^)
13^th^ Production unit^2^ # 149^th^ Regimental Farm, Shihezi city, Xinjiang (2016)	0–20	8.9	1.35	Sandy loam	53.8	18.8	207.6	15.4
	20–40	11.3	1.29					
	40–60	12.8	1.28					
	60–80	10.2	1.31					
11^th^ Production unit^2^ # 149^th^ Regimental Farm, Shihezi city, Xinjiang (2017)	0–20	9.2	1.33	Loam	61.7	21.8	213.2	16.8
	20–40	12.1	1.28					
	40–60	13.5	1.32					
	60–80	11.2	1.35					
Experimental farm # Shihezi University, Shihezi city, Xinjiang (2018)	0–20	12.9	1.42	Gray desert	54.9	19.1	194.2	15.6
	20–40	13.8	1.28					
	40–60	13.3	1.41					
	60–80	13.4	1.43					
Wulanwusu Agrometeorological Experiment Station # Shihezi city, Xinjiang (2019)	0–20	13.5	1.38	Loam	58.9	21.1	188.7	15.3
	20–40	14.4	1.31					
	40–60	15	1.29					
	60–80	13.8	1.38					

**Figure 2:**
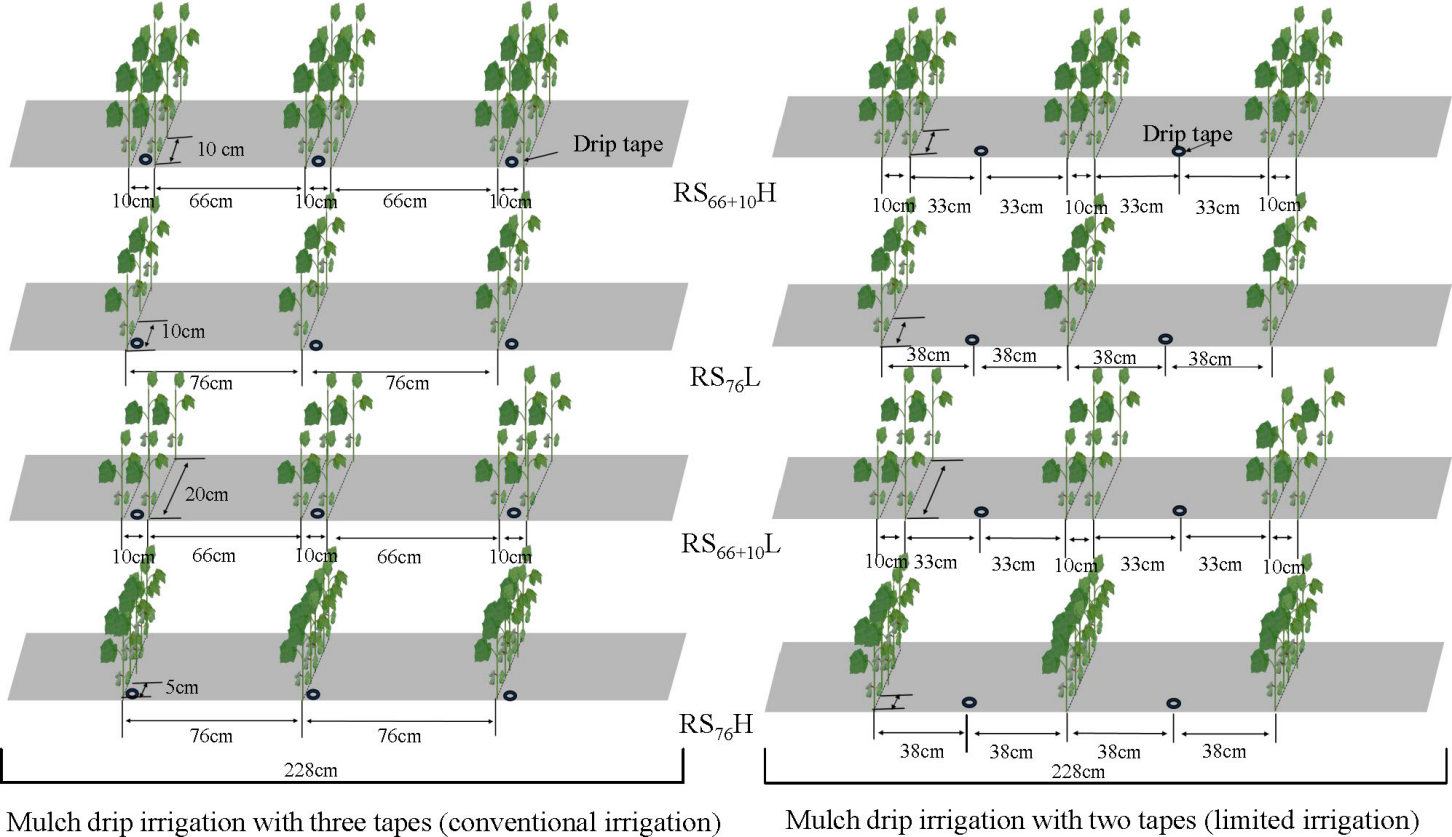
Schematic of four different planting densities of cotton (*Gossypium hirsutum*) evaluated during this study on cotton cultivation in Xinjiang, China. Where RS_66+10_H has 66+10 cm row spacing with 26 plants m^-2^ (high density), RS_76_H, where 76 cm row spacing with 26 plants m^-2^ (high density), RS_76_L has 76 cm row spacing with 13 plants m^-2^ (low density), and RS_66+10_L (66+10 cm row spacing with 13 plants m^-2^ (low density).

The experiment (two irrigation amount and four row spacing configurations) was arranged in a randomized complete block design with four replicates. Two irrigation amounts were applied to the main plots: the local conventional irrigation amount (CI) ranging from 510 to 600 mm adopting one film with three drip tapes, and limited irrigation (LI) adopting one film with two drip tapes (70% of the CI amount). This design was selected because large–scale drip irrigation cotton fields in Xinjiang adopt a rotation irrigation system, and the arrangement of one film with three-tapes as irrigation method has a greater water output per unit time, saving irrigation time and shortening the rotation cycle. The four row spacing configurations combined with planting density and row spacing were as follows: RS_66 + 10_H (66 + 10 cm row spacing with 26 plants m^−2^; high density), RS_76_H (76 cm row spacing with 26 plants m^−2^; high density), RS_76_L (76 cm row spacing with 13 plants m^−2^; low density), and RS_66 + 10_L (66 + 10 cm row spacing with 13 plants m^−2^; low density). Each subplot consisted of 12 (66 + 10 cm row spacing configuration) or 6 (76 cm row spacing configuration) ×10 m cotton plant rows with two 2.28 m – wide sheets of transparent plastic film (**Figure 2**). The diameter of the labyrinth drip irrigation tape was 12.5 mm; dripper flow rate was 2.2 m^3^ h^−1^, and dripper spacing was 20 cm.

The test sites were set up in the fields of local farmers, and each treatment consisting of one film with two tapes had a fertilizer amount consistent with that of the treatment consisting of one film with three tapes by adding fertilization tanks. Except for uniform seedling watering, all other irrigation periods were applied following a rotational irrigation system. Drip irrigation was applied 8–10 times during the growth period (**Table 2**).

**Table 2:** Sowing date, harvest period, total irrigation amount, and fertilization amount for different drip tape configurations in 2016, 2017, 2018, and 2019.

Year	Treatment	Seeding date (m/d)	Harvest date (m/d)	Irrigation amount (mm)	Fertilizer application (kg hm^−2^)		
					N	P	K
2016	CI	4/9	10/2	600	282.0	68.3	86.2
	LI	4/9	10/2	430	282.0	68.3	86.2
2017	CI	4/17	10/7	580	272.6	62.6	79
	LI	4/17	10/7	424	272.6	62.6	79
2018	CI	4/20	10/15	510	244.4	56.9	71.8
	LI	4/20	10/15	350	244.4	56.9	71.8
2019	CI	4/21	10/12	518	270.3	63.6	80.4
	LI	4/21	10/12	355	270.3	63.6	80.4

### 2.2 Soil water content and crop water use

Soil sample were excavated at 20 cm intervals (up to 80 cm deep soil profiles) by using a soil corer for the soil water content (SWC) measurement (n = 3) in each experimental plot. The measurements were executed with a horizontal distance of 0, 19, and 38 cm from the cotton row at 1 d before sowing, 1 d before irrigation, 2 d after irrigation, and maturity. The samples were immediately weighed and then baked at 80°C in an oven to determine the soil moisture content (SMC). The specific calculation formula for the soil accumulation water consumption (SAWC, mm) for different soil layers as follows:


(1) $$SAWC=\sum \left(SWC_{i+1}-SWC_{i}\right)$$

(2) SWC=H×SMC×P

(3) $$SMC=\left(M_{0}-M_{1}\right)/M_{1}\times 100\%$$

SWC_i+1_ and SWC_i_ means the soil water storage of one day before next irrigation and two days after irrigation respectively, in an irrigation cycle. H (mm) means the thickness of the soil layer; P (g·cm^–3^) means the soil bulk density. M_0_ and M_1_means the wet weight of soil sample and the dried weight of soil sample, respectively.

The daily water consumption intensity (DWCI, mm d^−1^) was determined by using Eq. (3) according to Wang et al. (2021b), which was used to identify the water consumption in different stages of cotton growth as follows:


(3) DWCI=ET/ΔT

where ET is the phase water consumption (mm) during a given growth period, and ΔT is the duration (d) of a given growth period.

Total crop water consumption, namely the actual evapotranspiration (ETc, mm), was calculated during the growing season as follows:


(4) ETc=R+I-F-Q+ΔW

where ETc is the crop evapotranspiration; where ETc (mm), R (mm), I (mm), F, and Q are the crop evapotranspiration, precipitation, irrigation amount, surface runoff, and capillary rise, respectively; ΔW is the change in SWC (mm). Q is the capillary rising to root zone, which is negligible due to the groundwater table of over 8 m at the experimental site. F could also be ignored at the experimental site.

### 2.3 Canopy apparent photosynthesis/transpiration rate

The canopy apparent photosynthesis (CAP) and canopy apparent transpiration rates (CAT) were simultaneously measured using the assimilation chamber method (Reddy et al., 1995; Xie et al., 2010). The CO_2_ and H_2_O concentrations in the chamber were measured using a Li–840A Soil CO_2_ Flux System (LI–COR Inc., Lincoln, NE, USA). The measurements were made between 11:00 and 14:00 h on clear days immediately after determining PAR. The assimilation chamber (85 cm long × 75 cm wide × 125 cm high) was covered with acrylic film that transmitted more than 95% of the solar radiation. Two fans were installed inside the chamber to mix the air. Gas exchange rates in each plot were measured during at least three 60 s intervals. We began to record the values when the CO_2_ concentrations inside the chamber began to drop steadily. Measurements were repeated three times for each treatment. The CAP and CAT calculation formula is as follows:


(5) $$CAP=\Delta C_{1}/10^{-6}\times V\times 360/\Delta M\times 273/\left(273+T\right)\times 44/22.4\times 1000/L$$

(6) $$CAT=\Delta C_{2}/10^{-6}\times V\times 360/\Delta M\times 273/\left(273+T\right)\times 44/22.4\times 1000/L$$

where ΔC_1_ represents net photosynthetic assimilation CO_2_ concentration in a given time interval (s); ΔC_2_ represents net photosynthetic assimilation H_2_O concentration in a given time interval (s); V is the assimilation chamber volume (m^3^); Δm is the measured time interval; T is the air temperature (°C); and L is the land area of the measured cotton canopy population.

### 2.4 Leaf area index

The leaf area index (LAI) was measured using the LAI–2200C canopy analyzer (Li–COR Inc., Lincoln, NE, USA) at 7–10 d intervals starting from early July, which referring to the method of Malone et al. (2002). Four to six readings were made in each plot. One measurement was made above the canopy, and then four measurements were made perpendicular to the cotton rows at the soil surface.

### 2.5 Dry matter accumulation and yield

For individual plant measurements, cotton plants were randomly selected in each plot at the initial flowering stage, full flowering stage, boll stage, and boll opening stage. On each sample date, four plants at each plot were randomly selected to obtain an average value. Plants were divided into various organs including stems, leaves, buds, and bolls. These segments were subsequently placed in paper bags, dried at 80 °C in an oven until constant weight, and the dry weight was measured. Daily dry matter accumulation was calculated as follows:


(7) $$DDMA=\left(DMA_{i+1}-DMA_{i}\right)/\Delta T$$

where DMA_i+1_ is the dried matter accumulation taken in the next growth period (g·m^−2^·d^−1^), and ΔT is the interval time for selecting dry matter accumulation. Seed cotton was hand harvested at 3 × 2.28 m^2^ area (n=4) in each plot at maturity. All mature cotton bolls in the 2.28*3 area are collected before harvest to facilitate data veracity. Seed cotton yield (kg hm^−2^) was determined for each plot after sun–drying for fifteen days, and then weighed after ginning to obtain the lint yield (kg hm^−2^).

### 2.6 Statistical analysis

Random block analysis of variance (ANOVA) was used to assess the effects of irrigation amount and row spacing configurations on LAI, CAP, CAT, ET_C_, DDMA, DWCI and seed/lint yield. Duncan’s multiple range tests were used to separate the treatment means at P< 0.05. Correlation analysis was conducted among LAI and CAP, CAT, DWCI, ET_C_, seed yield; DDMA and seed yield. Figures were constructed using the “lme4” and “ggplot2” packages in R 4.0.5 software (R Core Team 2021) and Sigmaplot 12.0 (Aspire Software Intl., Ashburn, USA).

## 3 Results

### 3.1 Cotton yield, and water use efficiency

The irrigation amount and row spacing configuration significantly affected daily dry matter accumulation (DDMA), seed yield, ET_C_, and WUE of cotton (*P*< 0.05; **Tables 3**; **4**). The DDMA under CI was 9.0–51.4% [before full flowering (BFF) – full flowering (FF)], 10.5–50.0% [FF – full boll stage (FB)] and 19.1–49.1% [FB –boll opening stage (BO)] greater than that of the same treatment under LI (*P*< 0.05). The seed yields, lint yields, and ET_C_ under CI were 6.6–18.3%, 7.8–14.3% and 22.9–32.6% higher, respectively, but the WUE was 6.2–19.0% lower than that of the same treatment under LI. The seed and lint yields of RS_66 + 10_H were 3.7–6.0% and 4.6–6.9% higher than those of RS_76_L under LI, respectively, while there was no significant difference in WUE (*P* > 0.05). Notably, under CI, the seed and lint yields of RS_76_L were not different from those of RS_66 + 10_H (*P* > 0.05), but ET_C_ was reduced by 51–60 mm and WUE was increased by 5.6–8.3% compared to RS_66 + 10_H

**Table 3:** Daily dry matter accumulation (DDMA) characteristics as affected by the combination of irrigation amount and row spacing configuration of cotton under mulch drip irrigation.

Irrigation amount	Row spacing	Daily dry matter accumulation (DDMA, g·m^−2^ d^−1^)											
		2016			2017			2018			2019		
		BFF-FF	FF-FB	FB-BO	BFF-FF	FF-FB	FB-BO	BFF-FF	FF-FB	FB-BO	BFF-FF	FF-FB	FB-BO
CI	RS_66 + 10_H	24.5a	37.2a	27.9a	29.9a	38.0a	30.5a	27.3a	37.8a	30.2a	29.7a	34.8a	28.1a
	RS_76_H	24.2a	36.5a	28.1a	28.3a	39.6a	27.6ab	28.6a	36.8a	29.0a	28.5a	32.9a	27.1a
	RS_76_L	22.4bc	32.0b	24.2bc	26.4b	35.5b	24.2c	24.3b	34.3b	26.5b	26.9b	30.2b	24.9b
	RS_66 + 10_L	23.3b	34.5ab	25.5b	25.5b	36.9b	24.9c	25.2b	35.1b	26.6b	27.3b	30.9b	25.3b
LI	RS_66 + 10_H	17.9de	26.9c	22.7cd	26.4b	29.1c	22.4d	23.4bc	34.0bc	24.3bc	26.1b	28.7bc	22.0c
	RS_76_H	19.2d	27.5c	23.1c	25.9b	30.1c	21.1d	23.0bc	33.3bc	24.6bc	26.3b	27.7c	21.8c
	RS_76_L	14.8f	21.7de	17.9e	23.0c	27.2cb	18.1e	21.7d	30.6d	21.8d	23.3c	25.8d	20.9d
	RS_66 + 10_L	15.9f	23.0d	17.1e	23.4c	26.8c	19.0e	21.1d	31.8d	21.5d	25.0c	27.0d	21.0d
I (Irrigation amount)		**	**	**	**	**	**	**	**	**	**	**	**
R (row)		NS	**	NS	**	**	**	**	NS	**	**	**	**
D (Density)		**	**	**	**	**	**	**	**	**	**	**	**
I×R		NS	NS	NS	**	**	**	**	**	**	**	**	**
I×D		NS	*	*	**	**	**	**	NS	**	**	**	**
R×D		**	**	**	**	**	**	**	**	**	**	**	**
I×R×D		NS	*	**	**	NS	**	**	NS	**	**	**	**

BFF, FF, FB, and BO means before full flowering, full flowering, full boll and boll opening of cotton growth stage, respectively. I means irrigation amount; R means row spacing configuration; D means plant density; CI means conventional irrigation; LI means limited irrigation; RS_66 + 10_H and RS_66 + 10_L mean high/low-density planting with 66 + 10 cm row spacing configuration, respectively; RS_76_H and RS_76_L mean high/low-density planting with 76 cm row spacing configuration, respectively. Values are means ± SD (n=4).* Significant at P ≤ 0.05; ** Significant at P ≤ 0.01; NS, not significant.

Values followed by different lowercase letters are significantly different at the 0.05 probability level.

**Table 4:** Seed yield, lint yield, crop evapotranspiration, and water use sufficiency as affected by the combination of irrigation amount and row spacing configuration of cotton under mulch drip irrigation.

Irrigation amount	Row spacing	Seed yield (kg·hm^−2^)				Crop evapotranspiration (ETc, mm)				Water use efficiency (kg·m^−3^)				Lint yield (kg·hm^−2^)
		2016	2017	2018	2019	2016	2017	2018	2019	2016	2017	2018	2019	
CI	RS_66 + 10_H	7358 ± 109a	7814 ± 85a	7576 ± 87a	7362 ± 80a	683 ± 10a	627 ± 12a	623 ± 13a	619 ± 6a	1.08 ± 0.02c	1.25 ± 0.01c	1.22 ± 0.01c	1.19 ± 0.01d	3389 ± 178a
	RS_76_H	7173 ± 175ab	7683 ± 63ab	7490 ± 73a	7289 ± 73a	681 ± 12a	631 ± 11a	624 ± 10a	621 ± 10a	1.05 ± 0.03c	1.22 ± 0.01cd	1.20 ± 0.01cd	1.17 ± 0.01d	3216 ± 203ab
	RS_76_L	7249 ± 14a	7711 ± 79a	7470 ± 20a	7276 ± 59a	623 ± 13b	571 ± 21bc	572 ± 17bc	568 ± 11cd	1.16 ± 0.00b	1.34 ± 0.01b	1.29 ± 0.00b	1.27 ± 0.01b	3315 ± 182a
	RS_66 + 10_L	7135 ± 156b	7573 ± 75b	7369 ± 30b	7207 ± 92a	639 ± 17b	591 ± 12b	595 ± 13b	582 ± 12c	1.12 ± 0.03b	1.26 ± 0.01c	1.23 ± 0.01c	1.24 ± 0.02bc	3173 ± 109ab
LI	RS_66 + 10_H	6846 ± 37c	6866 ± 35c	6898 ± 38c	6881 ± 62c	531 ± 12c	484 ± 13d	477 ± 11d	467 ± 11e	1.25 ± 0.01a	1.40 ± 0.01b	1.44 ± 0.02a	1.47 ± 0.01a	3021 ± 81c
	RS_76_H	6689 ± 138cd	6732 ± 62cd	6930 ± 95c	6841 ± 127c	528 ± 18c	486 ± 13d	477 ± 10d	472 ± 9e	1.27 ± 0.03a	1.39 ± 0.01b	1.45 ± 0.01a	1.45 ± 0.02a	2986 ± 156c
	RS_76_L	6468 ± 48d	6589 ± 74d	6731 ± 96d	6606 ± 42d	507 ± 8d	455 ± 17 e	460 ± 13de	450 ± 11f	1.28 ± 0.01a	1.46 ± 0.01a	1.46 ± 0.02a	1.47 ± 0.01a	2826 ± 88d
	RS_66 + 10_L	6519 ± 57cd	6318 ± 85e	6728 ± 14d	6623 ± 74d	520 ± 12cd	469 ± 12de	467 ± 14d	452 ± 15f	1.25 ± 0.01a	1.35 ± 0.02b	1.44 ± 0.00a	1.47 ± 0.02a	2810 ± 20
I (Irrigation amount)		**	**	**	**	**	**	**	**	**	**	**	**	**
R (row)		*	*	NS	NS	**	**	**	**	*	**	**	*	*
D (Density)		NS	**	NS	NS	**	**	**	**	*	**	**	*	NS
I×R		NS	NS	*	NS	NS	NS	**	**	*	NS	**	**	*
I×D		NS	NS	NS	NS	NS	NS	*	NS	NS	*	NS	NS	*
R×D		**	**	**	**	**	**	**	**	**	**	**	**	**
I×R×D		NS	*	NS	NS	**	**	**	**	**	**	**	**	NS

Lint yield data presented is a four-year average; CI means conventional irrigation; LI means limited irrigation; RS_66 + 10_H and RS_66 + 10_L mean high/low-density planting with 66 + 10 cm row spacing configuration, respectively; RS_76_H and RS_76_L mean high/low-density planting with 76 cm row spacing configuration, respectively. I means irrigation amount; R means row spacing configuration; D means plant density. Values are means ± SD (n=4).* Significant at P ≤ 0.05; ** Significant at P ≤ 0.01; NS, not significant.

Values followed by different lowercase letters are significantly different at the 0.05 probability level.

### 3.2 Soil accumulated water consumption in different soil layers

**Figure 3:**
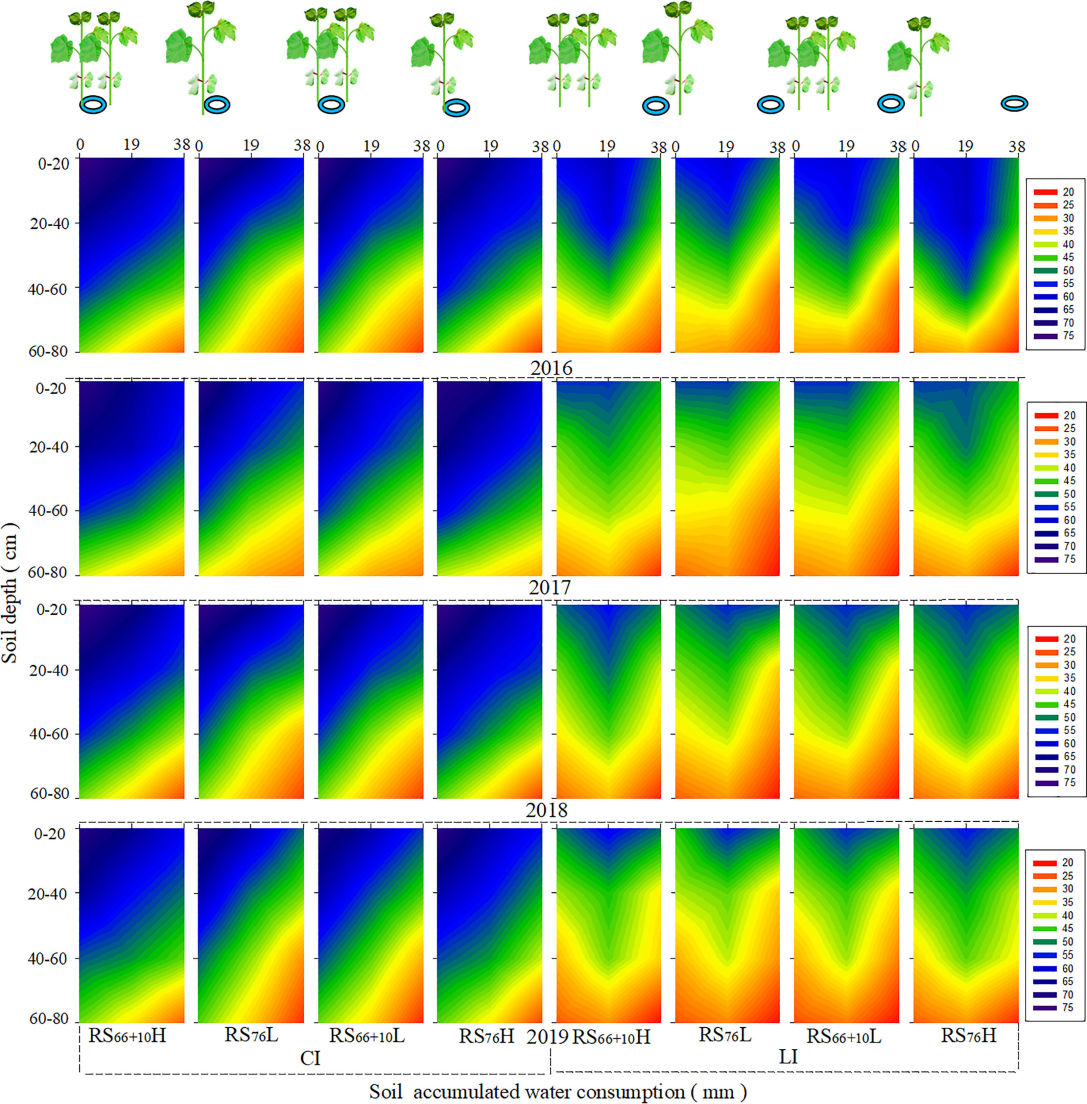
Soil accumulated water consumption (SAWC) in different soil layers under a combination of irrigation amount and row spacing configuration. The color changes from red to dark blue indicate a gradual increase in water consumption.

Under CI (**Figure 3**), the drip tapes were placed close to the cotton rows, and the trend of SAWC variation in each soil layer at a horizontal distance of 0–38 cm from the cotton row was in the following order:0 cm > 19 cm> 38 cm. Under LI, the trend variation of SAWC in each soil layer at 0–38 cm horizontal distance from the cotton rows was19 cm > 0 cm > 38 cm because the drip tapes were placed in the middle of the wide rows. The SAWC of the 0 –60 cm soil layer within 0, 19, and 38 cm radius from the horizontal distance of cotton rows under CI increased by 40.7–45.4%, 15.2–18.1% and 27.5–32.7% when compared to the same row spacing configuration under LI (*P<* 0.05). The highest SAWC was found in each soil layer in RS6_6_ + _10_H under the same irrigation amount (no difference between RS_66 + 10_H and RS_76_H). Under LI, the SAWC of RS_66 + 10_H was 4.2–6.9% and 6.8–11.4% greater than that of RS_76_L in the 20 – 40 cm and 40 – 60 cm soils at 19 cm from the horizontal distance of the cotton rows, respectively, and 10.6–11.1% and 13.1–13.2% greater than RS_76_L in the 20–40 cm and 40–60 cm soil layers at 38 cm from the horizontal distance of the cotton rows, respectively. Under CI, the SAWC of RS_76_L was 9.8–12.4% and 10.8–15.7% lower than RS_66 + 10_H in the 20–40 cm and 40–60 cm soil layers at a horizontal distance of 19 cm from the cotton rows, respectively: 7.7–16.8% and 9.1%–14.8% lower than RS_66 + 10_H in the 20–40 cm and 40–60 cm soil layers at a horizontal distance of 38 cm from the cotton rows, respectively.

### 3.3 Daily water consumption intensity and canopy apparent transpiration rate

The DWCI of cotton was significantly affected by the irrigation amount and row spacing configuration (**Table 5**, *P*< 0.05). The DWCI under CI was 11.5–30.2% (FF), 6.1–50.1%(FB) and 56.0–106.1%(BO) higher than that of the same row spacing under LI. The DWCI of RS_76_L was significantly lower than that of RS_66 + 10_H and RS_76_H under the same irrigation level, especially the largest difference during BO, where RS_76_L was 6.5–20.5% and 3.1–12.6% lower than RS_76_H and RS_66 + 10_H, respectively.

**Table 5:** Daily water consumption intensity as affected by the combination of irrigation amount and row spacing configuration of cotton under mulch drip irrigation.

Irrigation amount	Planting pattern	Daily water consumption intensity (DWCI, mmd^−1^)											
		2016			2017			2018			2019		
		FF	FB	BO	FF	FB	BO	FF	FB	BO	FF	FB	BO
CI	RS_66 + 10_H	5.7a	8.2a	3.1a	6.3a	8.1a	2.5a	6.7a	7.2a	3.1a	6.4a	7.4a	3.1a
	RS_76_H	5.9a	8.3a	3.1a	6.3a	8.0a	2.6a	6.6a	7.4a	3.2a	6.5a	7.4a	3.2a
	RS_76_L	5.2b	7.5b	2.7b	5.8c	7.4c	2.2b	6.1b	6.6b	2.9b	5.9b	6.9b	2.9ab
	RS_66 + 10_L	5.3ab	7.8ab	2.9ab	6.0ab	7.7ab	2.3ab	6.3ab	6.8ab	3.0ab	6.0ab	7.1ab	3.0a
LI	RS_66 + 10_H	4.9bc	7.5b	1.8b	4.9d	6.6d	1.3c	5.2c	5.0c	1.8c	5.0c	5.5c	2.0c
	RS_76_H	5.0b	7.6b	1.7b	4.9d	6.5d	1.3c	5.3c	4.9c	1.7c	5.1c	5.6c	1.9c
	RS_76_L	4.7c	7.1bc	1.5bc	4.6cd	6.2cd	1.2cd	4.8d	4.6d	1.6d	4.7cd	5.2cd	1.7cd
	RS_66 + 10_L	4.7c	7.3bc	1.6bc	4.7cd	6.3cd	1.3c	4.9cd	4.7cd	1.7cd	5.0c	5.2cd	1.8cd
I (Irrigation amount)		**	**	**	**	**	**	**	**	**	**	**	**
R (row)		**	**	NS	**	**	NS	**	**	NS	**	NS	**
D (Density)		NS	NS	NS	**	**	**	**	**	*	**	**	**
I×R		**	NS	**	NS	**	**	**	**	*	**	**	**
I×D		**	NS	NS	**	**	**	NS	**	NS	**	NS	**
R×D		**	**	**	**	**	**	**	**	**	**	**	**

FF, FB, and BO mean full flowering, full boll and boll opening of cotton growth stage, respectively. CI means conventional irrigation; LI means limited irrigation; RS_66 + 10_H and RS_66 + 10_L mean high/low-density planting with 66 + 10 cm row spacing configuration, respectively; RS_76_H and RS_76_L mean high/low-density planting with 76 cm row spacing configuration, respectively. I means irrigation amount; R means row spacing configuration; D means plant density. Values are means ± SD (n=4).* Significant at P ≤ 0.05; ** Significant at P ≤ 0.01; NS, not significant.

Values followed by different lowercase letters are significantly different at the 0.05 probability level.

**Figure 4:**
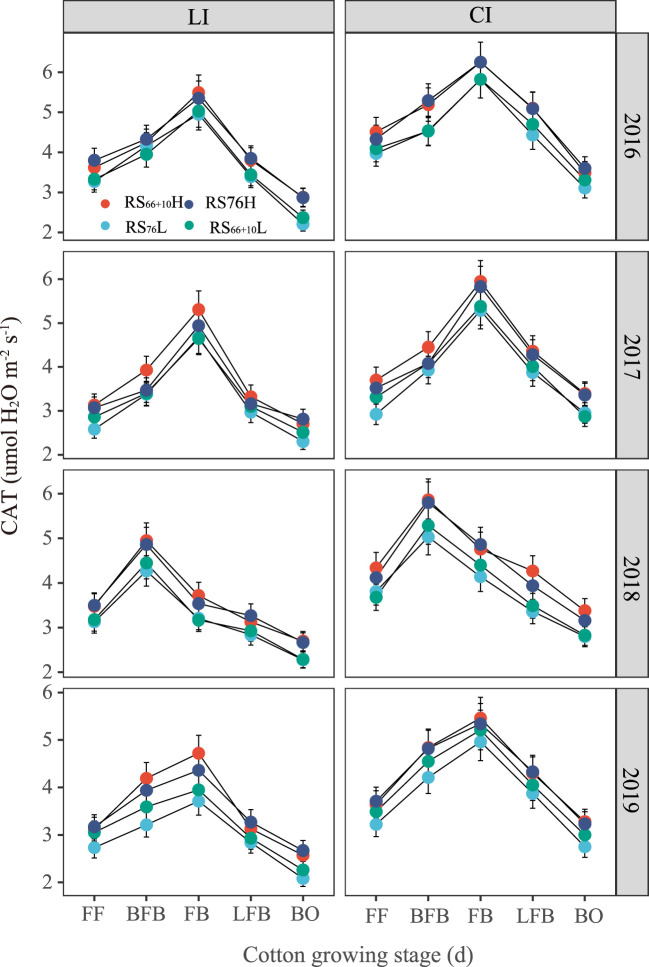
Canopy apparent transpiration rate (CAT) affected by the combination of irrigation amount and row spacing configuration. FF, BFB, FB, LFB and BO means full flowering, before full boll, full boll, later full boll and boll opening stage, respectively. Vertical bars represent the standard error. Mean values ± SE are from four replicates.

The CAT of cotton under CI was significantly higher (*P<* 0.05; **Figure 4**) than that of cotton with the same row spacing configuration under LI, especially 11.9–33.9% higher in FB. RS_76_L had the lowest CAT activity in all fertility periods under the same irrigation amount. Under LI, the CAT of RS_66 + 10_H was 9.2–23.5% and 3.8–22.3% higher than that of RS_76_L. Under CI, The CAT of RS_76_L was 10.1–30.6% and 3.9–22.2% lower than that of RS_66 + 10_H and RS_76_H, respectively, throughout the critical reproductive period.

### 3.4 Canopy apparent photosynthesis

The irrigation amount was found to significantly affect cotton CAP (*P<* 0.05; **Table 6**). The CAP under CI was 5.2–16.7%(FF), 8.8–23.4%(FB), and 20.7–71.6% (BO) higher than that under LI. RS_66 + 10_H had the largest CAP from FF to BO under the same irrigation amount. Under LI, RS_66 + 10_H had 2.8–5.1% and 19.8–45.7% higher CAP than RS_76_L in FB and BO, respectively. However, the CAP of RS_76_L was not significantly different from that of RS_66 + 10_H under CI (*P* > 0.05).

**Table 6:** Canopy apparent photosynthesis as affected by the combination of irrigation amount and row spacing configuration of cotton under mulch drip irrigation.

Irrigation amount	Planting pattern	Canopy apparent photosynthesis (CAP, μ mol m^−2^s^−1^)											
		2016			2017			2018			2019		
		FF	FB	BO	FF	FB	BO	FF	FB	BO	FF	FB	BO
CI	RS_66 + 10_H	30.3ab	37.3a	15.4a	30.4a	36.3a	14.0a	31.8a	30.8a	18.6a	29.2a	35.7a	18.3a
	RS_76_H	30.6ab	35.7ab	13.7ab	29.6a	34.5ab	13.5ab	30.5ab	28.9ab	17.3ab	28.1ab	36.4a	16.2b
	RS_76_L	31.4a	36.0ab	12.6bc	29.3a	34.2ab	13.7a	31.1ab	28.4ab	17.6ab	29.0a	35.3ab	17.1ab
	RS_66 + 10_L	32.9a	36.2ab	13.9ab	28.3ab	35.0ab	12.8b	32.5a	29.0ab	17.1ab	29.3a	34.5b	15.9b
LI	RS_66 + 10_H	28.6c	33.1c	10.9c	26.9cd	30.2cd	11.6c	30.4b	24.8c	15.3c	28.5ab	32.4cd	14.0c
	RS_76_H	27.6c	32.3c	11.1c	25.9d	31.7c	10.6c	29.0c	23.8cd	13.2d	27.5b	33.4c	13.2d
	RS_76_L	28.3c	31.7d	9.1d	25.9d	29.5d	9.0cd	30.2b	24.6c	10.5e	27.2b	30.9e	11.2e
	RS_66 + 10_L	28.2c	32.3c	8.1de	27.6c	30.9c	8.3d	29.7bc	23.5cd	11.0e	28.5ab	31.5d	10.2d
I (Irrigation amount)		**	**	**	**	**	**	**	**	**	**	**	**
R (row)		**	**	**	**	**	**	**	**	**	**	**	**
D (Density)		**	**	**	**	**	**	**	**	**	**	**	**
I×R		**	**	**	**	**	**	**	**	**	**	**	**
I×D		**	**	**	**	**	NS	**	**	**	**	**	**
R×D		**	**	**	**	**	**	**	**	**	**	**	**

FF, FB, and BO mean full flowering, full boll and boll opening of cotton growth stage, respectively. CI means conventional irrigation; LI means limited irrigation; RS_66 + 10_H and RS_66 + 10_L mean high/low-density planting with 66 + 10 cm row spacing configuration, respectively; RS_76_H and RS_76_L mean high/low-density planting with 76 cm row spacing configuration, respectively. I means irrigation amount; R means row spacing configuration; D means plant density. Values are means ± SD (n=4).* Significant at P ≤ 0.05; ** Significant at P ≤ 0.01; NS, not significant.

Values followed by different lowercase letters are significantly different at the 0.05 probability level.

### 3.5 Leaf area index

**Figure 5:**
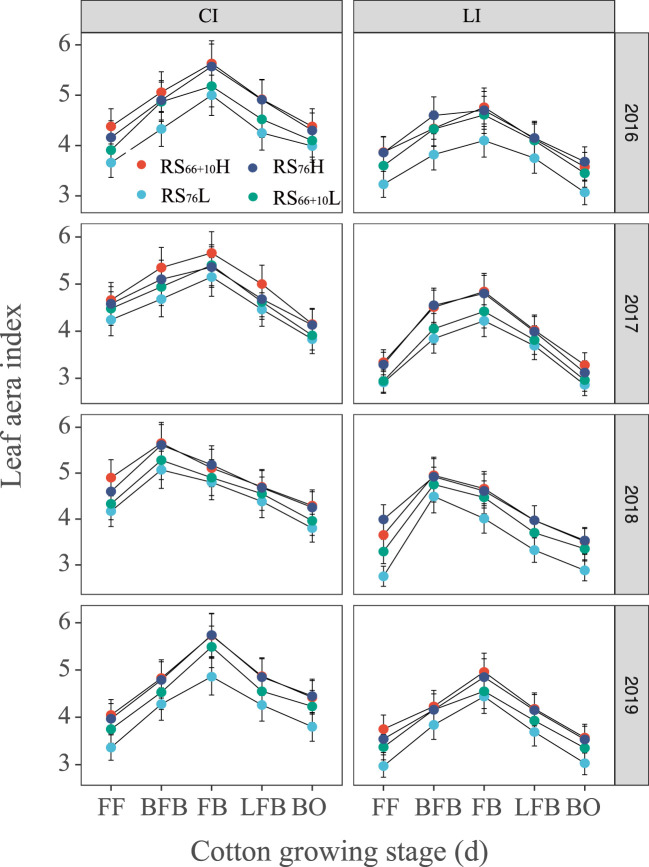
Leaf are index (LAI) of cotton in cotton (*Gossypium hirsutum*) affected by the combination of irrigation amount and row spacing configuration when evaluated in Xinjiang, China. FF, BFB, FB, LFB and BO means full flowering, before full boll, full boll, later full boll, and boll opening stage, respectively. Vertical bars represent the standard error. Mean values ± SE are from four replicates.

The LAI under CI was significantly greater than that under LI and was 9.5–22.2% greater at the FB (**Figure 5**). Under the same irrigation level, the trend variation of cotton LAI was RS_66 + 10_H, RS_76_H > RS_66 + 10_L > RS_76_L. The LAI of RS_66 + 10_H under LI was 5.1–25.8% and 7.8–32.3% higher than that of RS_76_L throughout the critical reproductive period. Under CI, RS_76_L was 6.0–17.1% and 7.3–16.8% lower than RS_66 + 10_H and RS_76_H, respectively, throughout the critical reproductive period from four replicates.

### 3.6 Relationship between LAI, yield and water consumption

**Figure 6:**
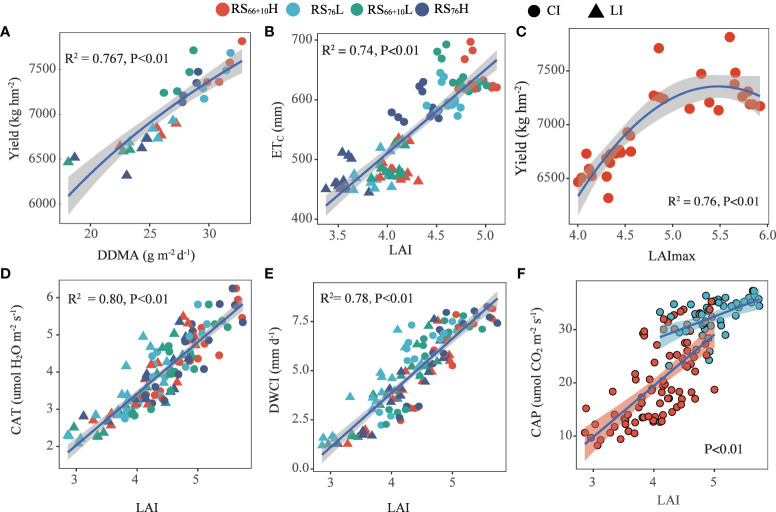
Correlation between leaf area index (LAI) and canopy apparent transpiration rate (CAT), daily water consumption intensity (DWCI), canopy apparent photosynthesis (CAP), crop evapotranspiration (ET_C_), and Yield, and the correlation between daily dry matter accumulation (DDMA) and Yield. DDMA **(A)**, ET_C_ **(B)** and LAI **(E, F)** were the mean values from full flowering to the boll opening stage; LAImax **(C)** is the maxmum LAI value in critical growth period. CAP **(F)**, CAT **(D)**, and DWCI **(E)** were the mean values of each growth period (full flowering stage, full boll stage, and boll opening stage) corresponding to LAI. CI (conventional irrigation); LI (limited irrigation); RS_66+10H_ and RS_66+10L_ (high/low-density planting with 66+10cm row spacing configuration); RS_76_H and RS_76_L (high/low-density planting with 76 cm row spacing configuration).

The relationship between LAI_max_ and seed yield was fitted to a quadratic function (*P*< 0.01; **Figure 6**), and the maximum cotton seed yield (7366 kg hm^–2^) was obtained when LAI_max_ was approximately 5.5. However, based on the correlation between LAI and CAP, after LAI_max_ reached 5, further increases in LAI did not significantly increase CAP, which indicated that a higher LAI (peak >5) did not significantly increase cotton CAP and seed yields. There was a highly significant positive correlation between CAT, DWCI, ETc, and LAI (*P*< 0.01). This indicates that the LAI is a principal factor affecting cotton transpiration water consumption under mulch drip irrigation. Therefore, combine maintaining cotton high yields and water conservation, the optimal cotton LAI_max_ range should be between 5.0 and 5.5 (**Figures 6C, F**).

## 4 Discussion

### 4.1 Leaf area index, photosynthetic rate, and cotton yield

The first objective of this study was to evaluate the effect of irrigation amount and row spacing configuration on LAI, photosynthesis, dry matter accumulation rate, and yield. Adjusting the planting density and row spacing configuration is an important agronomic measure for achieving high and stable cotton yields (Zhang et al., 2004; Brodrick et al., 2010; Chen et al., 2019). An important condition for achieving high cotton yields in cotton production areas with short frost–free periods and limited light and heat resources is to achieve a high rate of dry matter accumulation per unit area. This study showed that cotton DDMA was higher under CI than under the same row spacing configuration with LI (**Table 3**), indicating that higher dry matter accumulation rates and higher yields were obtained under CI conditions during shorter reproductive periods (**Figure 6A**). A suitable LAI range is essential for the rapid growth of cotton dry matter accumulation (Srinivasan et al., 2017). Hu et al. (2021) concluded that a larger LAI caused canopy shading between leaves, resulting in lower CAP and lower cotton yield levels. This study showed a highly significant quadratic relationship between cotton LAI_max_ and seed yield (**Figure 6C**). It had the highest yield when LAI_max_ reached approximately 5.4 (**Figure 5**). Analysis of the relationship between LAI and CAP (**Figure 6F**) showed that CAP increased significantly when 0< LAI_max_< 5. However, when LAI_max_ > 5, CAP did not increase significantly, which may be related to mutual shading between the groups. In combination with the CAP of treatments under CI, the cotton population maintained a relatively stable and high CAP when LAI_max_ between 5.0 and 6.0. This may be attributed to the adoption of densely tolerant cotton varieties in Xinjiang and the optimization of canopy structure through chemical regulation to shape compact plants (Wang et al., 2021a; Shi et al., 2022). However, RS_76_L under CI reached the same yield level as RS_66 + 10_H, however its LAI_max_ was between 5.0 and 5.5, which was significantly smaller than RS_66 + 10_H and RS_76_H. Combining LAI_max_, CAP, and cotton yield, the appropriate LAI_max_ for achieving a high cotton yield in Xinjiang was between 5.0 and 5.5.

The cotton regions of the Yellow River Basin and Yangtze River Basin in China have a long cotton fertility period. The suitable planting density was 50000–60000 plants hm^−2^ and the largest cotton seed yield was 3700–4500 kg hm^−2^ in the Yellow River basin (Li et al., 2020); The optimum density was 19500–37500 plants hm^−2^ and the largest seed yield was 3800–4200 kg hm^−2^ in the Yangtze River basin (Lv et al., 2021). Based on the literature, our analysis of the relationship between cotton LAI and CAP in the Yangtze and Yellow River basins revealed a quadratic relationship (**Figure S1**) and the optimum LAI_max_ was between 3.5 and 4.0. Therefore, in cotton areas with a short reproductive period, higher cotton population photosynthetic capacity and higher yield could be achieved by using a combination of adequate irrigation with appropriate low-density row spacing, or with high density to improve LAI under limited drip irrigation.

### 4.2 Optimal planting pattern of cotton is regulated by the local water resource condition

The second objective of the study was to integrate yield and soil water consumption to optimize the row spacing configuration under different irrigation conditions. The analysis showed that LAI was linearly and positively correlated with CAT, DWCI, and ET_C_ (**Figures 6D, E, B**), and we concluded that LAI was a key factor affecting the soil evaporation and transpiration of cotton. The cotton yields under CI were significantly higher than those under LI, but the ET_C_ increased by 22.9–32.6% under CI compared to those observed under LI, mainly because of the higher LAI. Therefore, CI is recommended to achieve higher yields in areas with sufficient water. Under CI, RS_76_L reduced CAT and DWCI because of lower LAI and overall reduced cotton ET_C_ and significantly increased WUE (**Table 4**, *P*< 0.05) relative to RS_66 + 10_H and RS_76_H under adequate irrigation. Both CAT and DWCI were significantly lower in RS_76_L than in RS_66 + 10_H and RS_76_H under the same irrigation amount (**Figure 4**; **Table 5**).

Planting density is also an important factor affecting crop ET_C_, and related studies have shown that an increased planting density of maize significantly increases ET_C_ (Guo et al., 2021). Deep soil water consumption is significantly elevated owing to the high planting density (Magaia et al., 2017; Meng et al., 2020). RS_76_L under CI significantly reduced SAWC in the 20–60 cm soil layer within a horizontal distance of 19–38 cm from the cotton row radius compared with RS_66 + 10_H and RS_76_H (**Figure 3**). High-density planting, such as RS_66 + 10_H, did not result in significant drought stress relative to RS_76_L under adequate irrigation. However, the ET_C_ of RS_76_L under CI decreased by 51–60 mm, but the WUE increased by 5.6–8.3% compared to RS_66 + 10_H, which indicated that RS_76_L could further reduce irrigation to improve water use efficiency under CI. In conjunction with the development of machine harvesting cotton in China, RS_76_L under adequate irrigation is more conducive to cotton defoliation than RS_66 + 10_H and RS_76_H because of the larger row spacing and lower LAI in late reproduction (Li et al., 2016; Hu et al., 2021), which reduced cotton seed inclusion and improved cotton quality after mechanical harvesting. Moreover, RS_76_L saved seed cost and cotton labor topping cost compared to RS_66 + 10_H, owing to half of the seeding volume. In summary, the combination of low-density equal row spacing with CI could reduce soil water consumption in the 20–60 cm soil layer while maintaining high cotton yields and has the potential to further reduce irrigation. Therefore, RS_76_L under CI is also conducive to improving the machine–harvested quality of cotton and reducing management costs and is an optimum cotton planting pattern for mulch drip irrigation in arid areas.

Under LI, RS_66 + 10_H had the highest cotton yield, but the WUE did not differ from that of RS_76_L and RS_66 + 10_L (*P* > 0.05). High-density planting of cotton under LI can make full use of deep soil water by increasing root length and root surface area, inducing root growth in the deep and lateral soil layers, and promoting water uptake and transport for normal aboveground growth and development (Dong et al., 2010; Chen et al., 2018). Our results showed that RS_66 + 10_H significantly increased soil water consumption in the 20–60 cm soil layer at a horizontal distance of 19 –38 cm from the cotton row compared with RS_76_L under LI. Because of the use of mulch drip irrigation, soil water and roots are mainly distributed in the 0–60 cm soil layer range (Wang et al., 2014; Chen et al., 2022). The distribution of cotton roots under high-density planting coincided with the water supply in the 20–60 cm soil layer, which was fully utilized. Therefore, in cotton production areas where water resources are scarce, high-density planting can be used to tap the soil water production potential to achieve high and stable cotton yields.

## 5 Conclusion

This study proposed the most suitable planting pattern based on different irrigation conditions. This research showed that a maximum LAI (LAI_max_) maintained between 5.0 and 5.5 was most conducive to high yield and higher WUE. Under sufficient water, optimize low density row spacing configuration (RS_76_L) could reach the same yield level as high-density planting, whereas suitable LAI_max_ reduced CAT, DWCI, and soil water consumption of 20-60 cm soil layers. Under water restriction condition, high-density planting (RS_66 + 10_H) could fully exploit the soil water potential of 20-60 cm soil layers to improve cotton yields. Our results suggest that in northern of Xinjiang’s moisture-rich areas, RS_76_L has the advantage of receiving high cotton yield while improving cotton benefits and further reducing the irrigation amount to improve WUE. However, moisture-limited areas are more suitable for high-density planting to increase yields.

## Data availability statement

The original contributions presented in the study are included in the article/**Supplementary Material**, further inquiries can be directed to the corresponding author.

## Author contributions

WQZ, BW and YW conceptualized the study. WQZ, WFZ and JT involved in methodology. WQZ and BW involved in formal analysis and writing— original draft. WQZ, XJ and HD investigated the study. WQZ and SX involved in writing—review and editing. WFZ supervised the study. All authors contributed to the article and approved the submitted version.

## References

[B1] Anwar M. R. Wang B. Li Liu D. Waters C. (2020). Late planting has great potential to mitigate the effects of future climate change on Australian rainfed cotton. Sci. Total Environ. 714, 136806. 31982770 10.1016/j.scitotenv.2020.136806

[B2] Araus J. L. Sanchez-Bragado R. Vicente R. (2021). Improving crop yield and resilience through optimization of photosynthesis: panacea or pipe dream? J. Exp. Bot. 72, 3936–3955. 33640973 10.1093/jxb/erab097

[B3] Bilal A. Ahmad A. Rasul F. Murtaza G. (2019). Optimization of the sowing time for bt-cotton production in punjab, pakistan. Pak. J. Biol. Agric. Sci. 56, 95–100.

[B4] Brodrick R. Bange M. P. Milroy S. P. Hammer G. L. (2010). Yield and maturity of ultra-narrow row cotton in high input production systems. Agron. J. 102 (3), 843–848.

[B5] Chen W. Chen F. Lai S. Jin M. Xu S. Liu Y.. (2022). Spatial distribution and dynamics of cotton fine root under film-mulched drip irrigation. Ind. Crops Prod. 179, 114693.

[B6] Chen Z. Niu Y. Zhao R. Han C. Han H. Luo H. (2019). The combination of limited irrigation and high plant density optimizes canopy structure and improves the water use efficiency of cotton. Agric. Water Manage. 218, 139–148.

[B7] Chen Z. Tao X. Khan A. Tan D. K. Luo H. (2018). Biomass accumulation, photo-synthetic traits and root development of cotton as affected by irrigation and nitrogen-fertilization. Front. Plant Sci. 9, 173–187. 29497435 10.3389/fpls.2018.00173 PMC5819319

[B8] Cui Z. Zhang H. Chen X. Zhang C. Ma W. Huang C.. (2018). Pursuing sustainable productivity with millions of smallholder farmers. Nature. 555, 363–366. 29513654 10.1038/nature25785

[B9] Dai J. Dong H. (2014). Intensive cotton farming technologies in China: achievements, challenges and countermeasures. Field Crops Res. 155, 99–110.

[B10] Di N. Wang Y. Clothier B. Liu Y. Jia L. Xi B.. (2019). Modeling soil evaporation and the response of the crop coefficient to leaf area index in mature populus tomentosa plantations growing under different soil water availabilities. Agric. For. Meteorol. 264, 125–137.

[B11] Dong H. Kong X. Luo Z. Li W. Xin C. (2010). Unequal salt distribution in the root zone increases growth and yield of cotton. Eur. J. Agron. 33, 285–292.

[B12] Dong H. Li W. Tang W. Li Z. Zhang D. Niu Y. (2006). Yield, quality and leaf senescence of cotton grown at varying planting dates and plant densities in the yellow river valley of China. Field Crops Res. 98, 106–115.

[B13] Feng L. Dai J. Tian L. Zhang H. Li W. Dong H. (2017). Review of the technology for high-yielding and efficient cotton cultivation in northwest inland cotton-growing region of China. Field Crops Res. 208, 18–26.

[B14] Forouzani M. Karami E. (2011). Agricultural water poverty index and sustainability. Agron. Sustain. Dev. 31, 415–431.

[B15] Guo Q. Huang G. Guo Y. Zhang M. Zhou Y. Duan L. (2021). Optimizing irrigation and planting density of spring maize under mulch drip irrigation system in the arid region of Northwest China. Field Crops Res. 266, 108141.

[B16] Heitholt J. J. (1994). Canopy characteristics associated with deficient and excessive cotton plant population densities. Crop Sci. 34, 1291–1297.

[B17] Hernandez M. D. Alfonso C. Echarte M. M. Cerrudo A. Echarte L. (2021). Maize transpiration efficiency increases with n supply or higher plant densities. Agric. Water Manage. 250, 106816.

[B18] Hou X. Fan J. Zhang F. Hu W. Yan F. Xiao C.. (2022). Determining water use and crop coefficients of drip-irrigated cotton in south xinjiang of China under various irrigation amounts. Ind. Crops Prod. 176, 114376.

[B19] Hu L. Pan X. Wang X. Hu Q. Wang X. Zhang H.. (2021). Cotton photosynthetic productivity enhancement through uniform row-spacing with optimal plant density in xinjiang, China. Crop Sci. 61, 2745–2758. 34413536 10.1002/csc2.20535 PMC8361742

[B20] Kerby T. A. Cassman K. G. Keeley M. (1990). Genotypes and plant densities for narrow-row cotton systems. II. leaf area and dry-matter partitioning. Crop Sci. 30, 649–653.

[B21] Khan A. Najeeb U. Wang L. Tan D. K. Y. Yang G. Munsif F.. (2017b). Planting density and sowing date strongly influence growth and lint yield of cotton crops. Field Crops Res. 209, 129–135.

[B22] Khan A. Wang L. Ali S. Tung S. A. Hafeez A. Yang G. (2017a). Optimal planting density and sowing date can improve cotton yield by maintaining reproductive organ biomass and enhancing potassium uptake. Field Crops Res. 214, 164–174.

[B23] Kodur S. (2017). Improving the prediction of soil evaporation for different soil types under dryland cropping. Agric. Water Manag 193, 131–141.

[B24] Li J. F. Liang F. B. Chen H. C. Zhang W. F. Kang P. (2016). Effect of row spacing configuration on agronomic traits and yield of cotton under machine-harvested pattern. Xinjiang Agric. Sci. 08), 1390–1396.

[B25] Li H. Wan H. L. Tian L. W. Liu L. T. Zhang Y. J. Bai Z. Y.. (2020). The effects of increased-density on canopy apparent photosynthesis, dry matter accumulation and distribution of cotton under late-sown condition. Cotton Sci. 32, 339–347.

[B26] Li C. Wu P. T. Li X. L. Zhou T. W. Sun S. K. Wang Y. B.. (2017). Spatial and temporal evolution of climatic factors and its impacts on potential evapotranspiration in loess plateau of northern shaanxi, China. Sci. Total Environ. 589, 165–172. 28258753 10.1016/j.scitotenv.2017.02.122

[B27] Li H. Zhu Y. F. Cai D. L. (2021). Study on the characteristics of water use in agriculture and major crops in xinjiang. Agric. Technol. 41 (21), 40–43.

[B28] Liao Z. Zeng H. Fan J. Lai Z. Zhang C. Zhang F.. (2022). Effects of plant density, nitrogen rate and supplemental irrigation on photosynthesis, root growth, seed yield and water-nitrogen use efficiency of soybean under ridge-furrow plastic mulching. Agric. Water Manage. 268, 107688.

[B29] Luo H. H. Zhang H. Z. Han H. Y. Zhang Y. L. Zhang W. F. (2014). Effects of water sto-rage in deeper soil layers on growth, yield, and water productivity of cotton (Gossypium hirsutum l.) in arid areas of northwestern China. Irrig. Drain. 63, 59–70.

[B30] Lv X. Wang Z. Ma L. Cao N. Meng Y. Zhou Z. (2021). Crop residue incorporation combined with potassium fertilizer increased cotton canopy apparent photosynthesis and seed cotton yield in barley-cotton rotation system. Arch. Agron. Soil Sci. 67, 300–312.

[B31] Magaia E. Famba S. Wesström I. Brito R. Joel A. (2017). Modelling maize yield response to plant density and water and nitrogen supply in a semi-arid region. Field Crop Res. 205, 170–181.

[B32] Malone S. Herbert D. A. Holshouser D. L. (2002). Evaluation of the LAI-2000 plant canopy analyzer to estimate leaf area in manually defoliated soybean. Agron. J. 94, 1012–1019.

[B33] Meng X. Lian Y. Liu Q. Zhang P. Jia Z. Han Q. (2020). Optimizing the planting density under the ridge and furrow rainwater harvesting system to improve crop water productivity for foxtail millet in semiarid areas. Agric. Water Manage. 238, 106220.

[B34] Neumann K. Stehfest E. Verburg P. H. Siebert S. Müller C. Veldkamp T. (2011). Exploring global irrigation patterns: a multilevel modelling approach. Agr. Syst. 104, 703–713.

[B35] Paul M. Rajib A. Negahban-Azar M. Shirmohammadi A. Srivastava P. (2021). Improved agricultural water management in data-scarce semi-arid watersheds: value of integrating remotely sensed leaf area index in hydrological modeling. Sci. Total Environ. 791, 148177. 34118663 10.1016/j.scitotenv.2021.148177

[B36] Qin S. Li S. Kang S. Du T. Tong L. Ding R. (2016). Can the drip irrigation under film mulch reduce crop evapotranspiration and save water under the sufficient irrigation condition? Agric. Water Manage. 177, 128–137.

[B37] Rafiee M. Kalhor M. (2016). Economic water use efficiency of corn (Zea mays l.) hybrids as affected by irrigation regimes: a case study in West Iran. Arch. Agron. Soil Sci. 62, 781–789.

[B38] Rahman M. A. Moser A. Gold A. Rötzer T. Pauleit S. (2018). Vertical air temperature gradients under the shade of two contrasting urban tree species during different types of summer days. Sci. Total Environ. 633, 100–111. 29573677 10.1016/j.scitotenv.2018.03.168

[B39] Reddy V. Reddy K. Hodges H. (1995). Carbon dioxide enrichment and temperature effects on cotton canopy photosynthesis transpiration, and water-use effciency. Field Crops Res. 41, 13–23.

[B40] Shi F. Li N. N. Khan A. Lin H. R. Tian Y. Shi X.. (2022). DPC can inhibit cotton apical dominance and increase seed yield by affecting apical part structure and hormone content. Field Crop Res. 282, 108509.

[B41] Srinivasan V. Kumar P. Long S. P. (2017). Decreasing, not increasing, leaf area will raise crop yields under global atmospheric change. Glob. Change Biol. 23, 1626–1635. 10.1111/gcb.13526 PMC5347850 27860122

[B42] Sui J. Wang J. Gong S. Xu D. Zhang Y. Qin Q. (2018). Assessment of maize yield-increasing potential and optimum n level under mulched drip irrigation in the northeast of China. Field Crop Res. 215, 132–139.

[B43] Tabashnik B. E. Carrière Y. (2019). Global patterns of resistance to bt crops highlighting pink bollworm in the united states, China, and India. J. Econ. Entomol. 112, 2513–2523. 31254345 10.1093/jee/toz173

[B44] Tian J. Zhang X. Yang Y. Xu S. Zuo W. Zhang W.. (2017). How to reduce cotton fiber damage in the xinjiang China. Ind. Crops Prod. 109, 803–811.

[B45] Wang Y. Chen M. Liang F. Tian J. Zhang Y. Jiang C.. (2021a). Photosynthates competition within the boll–leaf system is alleviated with the improvement of photosynthetic performance during the succession of xinjiang cotton cultivars. Ind. Crops Prod. 160, 113121.

[B46] Wang F. Xiao J. Ming B. Xie R. Wang K. Hou P.. (2021b). Grain yields and evapotranspiration dynamics of drip-irrigated maize under high plant density across arid to semi-humid climates. Agric. Water Manage. 247, 106726.

[B47] Wang Z. Yang P. Zheng X. He X. Zhang J. Li W. (2014). Soil salt dynamics in cotton fields with mulched drip irrigation under the existing irrigation system in xinjiang. Agric. Machinery 45, 149–159.

[B48] Wei K. Zhang J. Wang Q. Guo Y. Mu W. (2022). Irrigation with ionized brackish water affects cotton yield and water use efficiency. Ind. Crops Prod. 175, 114244.

[B49] Wu Y. Huang F. Y. Jia Z. K. Ren X. L. Cai T. (2017). Response of soil water, temperature, and maize (Zea may l.) production to different plastic film mulching patterns in semi-arid areas of northwest China. Soil Tillage Res. 166, 113–121.

[B50] Xie T. T. Su P. X. Gao S. (2010). Photosynthetic rate, transpiration rate, and water use efficiency of cotton canopy in oasis edge of linze. J. Appl. Ecol. 21, 1425–1431. 20873616

[B51] Yao H. Zhang Y. Yi X. Zuo W. Lei Z. Sui L.. (2017). Characters in light-response curves of canopy photosynthetic use efficiency of light and n in responses to plant density in field-grown cotton. Field Crop Res. 203, 192–200.

[B52] Zhang W. Wang Z. Yu S. Li S. Fang J. Tong W. (2004). Effects of planting density on canopy photosynthesis, canopy structure and yield formation of high-yield cotton in xinjiang, China. Chin. J. Plant Ecol. 28, 164.

[B53] Zou H. Fan J. Zhang F. Xiang Y. Wu L. Yan S. (2020). Optimization of drip irrigation and fertilization regimes for high grain yield, crop water productivity and economic benefits of spring maize in Northwest China. Agric. Water Manage. 230, 105986.

