# Supplementary figures and images for: Optimal planting pattern of cotton is regulated by irrigation amount under mulch drip irrigation
Source: Front Plant Sci. 2023 May 30;14:1158329. doi: 10.3389/fpls.2023.1158329 (PMC10265678; doi:10.3389/fpls.2023.1158329)

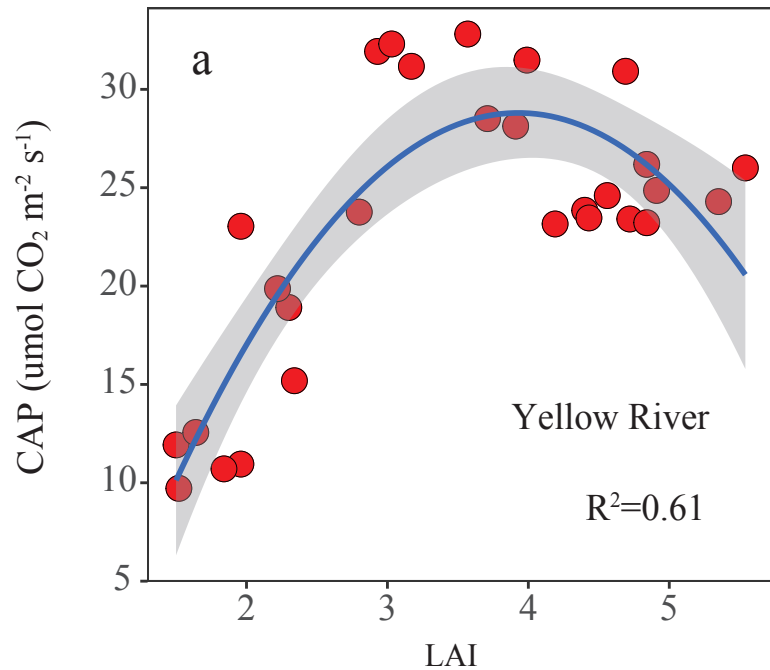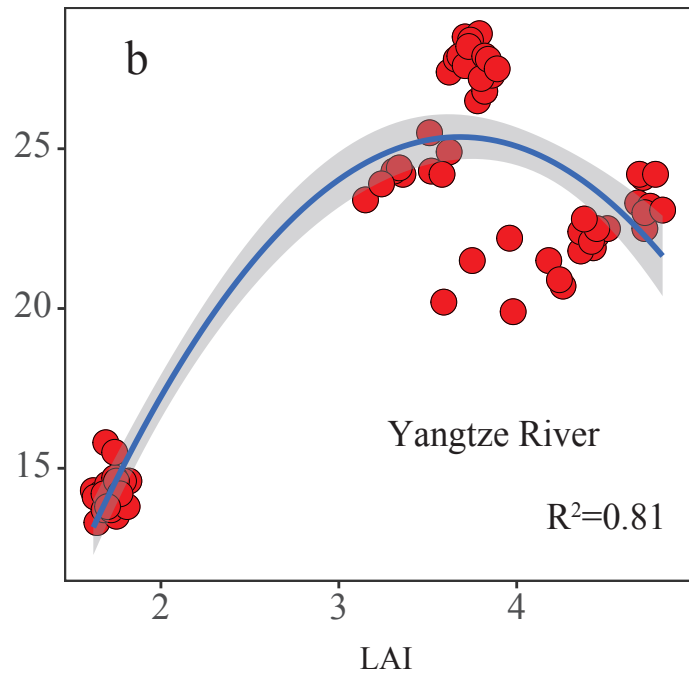

Supplement: Supplementary file 1 [file Image_1.pdf]
